# Zooplankton diversity analysis through single-gene sequencing of a community sample

**DOI:** 10.1186/1471-2164-10-438

**Published:** 2009-09-17

**Authors:** Ryuji J Machida, Yasuyuki Hashiguchi, Mutsumi Nishida, Shuhei Nishida

**Affiliations:** 1 Ocean Research Institute, University of Tokyo, 1-15-1 Minamidai, Nakano-ku, Tokyo 164-8639, Japan; 2 Department of Biology, Osaka Medical College, Daigaku-machi 2-7, Takatsuki, Osaka 569-8686, Japan

## Abstract

**Background:**

Oceans cover more than 70% of the earth's surface and are critical for the homeostasis of the environment. Among the components of the ocean ecosystem, zooplankton play vital roles in energy and matter transfer through the system. Despite their importance, understanding of zooplankton biodiversity is limited because of their fragile nature, small body size, and the large number of species from various taxonomic phyla. Here we present the results of single-gene zooplankton community analysis using a method that determines a large number of mitochondrial *COI *gene sequences from a bulk zooplankton sample. This approach will enable us to estimate the species richness of almost the entire zooplankton community.

**Results:**

A sample was collected from a depth of 721 m to the surface in the western equatorial Pacific off Pohnpei Island, Micronesia, with a plankton net equipped with a 2-m^2 ^mouth opening. A total of 1,336 mitochondrial *COI *gene sequences were determined from the cDNA library made from the sample. From the determined sequences, the occurrence of 189 species of zooplankton was estimated. BLASTN search results showed high degrees of similarity (>98%) between the query and database for 10 species, including holozooplankton and merozooplankton.

**Conclusion:**

In conjunction with the Census of Marine Zooplankton and Barcode of Life projects, single-gene zooplankton community analysis will be a powerful tool for estimating the species richness of zooplankton communities.

## Background

The fauna of the world's oceans is dominated in terms of abundance and biomass by drifting organisms collectively referred to as plankton. Plankton occur in all marine waters, throughout all depths, and, for many species, across widespread biogeographical regions. Zooplankton (planktonic animals) support many major fisheries and mediate fluxes of nutrients and chemical elements essential to life on earth. Despite more than a century of sampling the oceans, a comprehensive understanding of zooplankton biodiversity has eluded oceanographers because of the fragile nature and small body size of these organisms, as well as the large number of species from various taxonomic phyla [1,2]. For many zooplankton groups, there are longstanding and unresolved questions of species identification, systematic relationships, genetic diversity, and biogeography. In light of this, we are working toward a taxonomically comprehensive assessment of zooplankton biodiversity throughout the world's oceans through the international project Census of Marine Zooplankton [3].

## Results and Discussion

**Figure 1:**
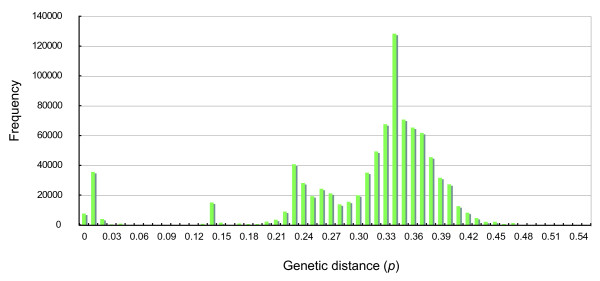
**Mismatch distributions of the pairwise genetic distances for the 1,336 mitochondrial *COI *sequences**. A total of 891,780 frequencies calculated from the 1,336 sequences are plotted in the figure.

**Figure 2:**
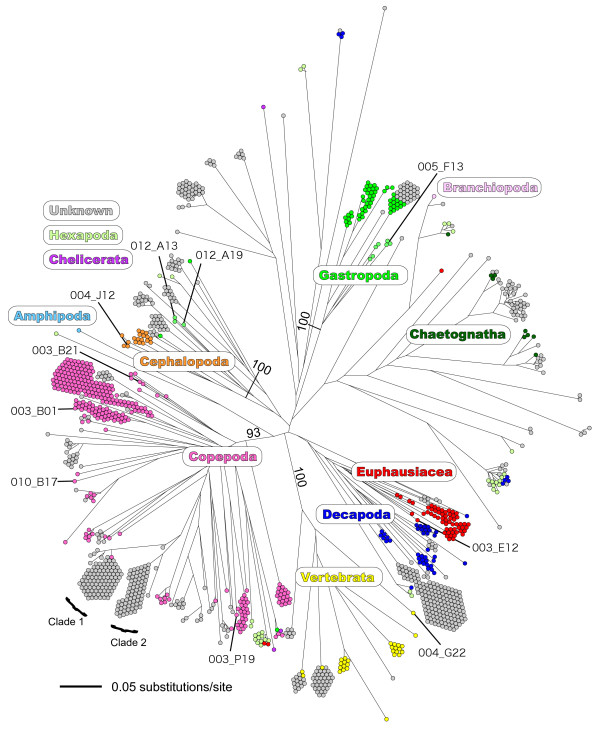
**Unrooted neighbour-joining tree of the 1,336 mitochondrial *COI *gene sequence**. Numbers beside internal branches indicate bootstrap values (>90%) obtained for 1,000 replicates (indicated for major branches only). Each dot represents a single mitochondrial *COI *gene sequence. Colour of each dot represent the higher taxonomic groups denoted in the figure with the criterion of the score and similarity more than 100 and 83%, respectively, in the BLAST results.

**Figure 3:**
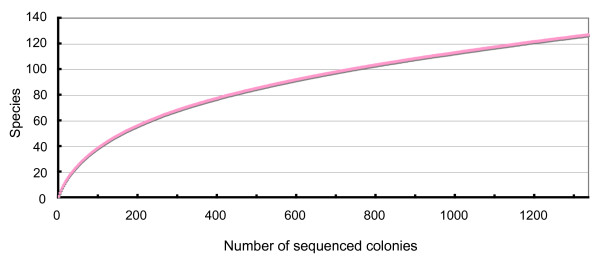
**Rarefaction analysis of the 1,336 mitochondrial *COI *gene sequences**.

**Figure 4:**
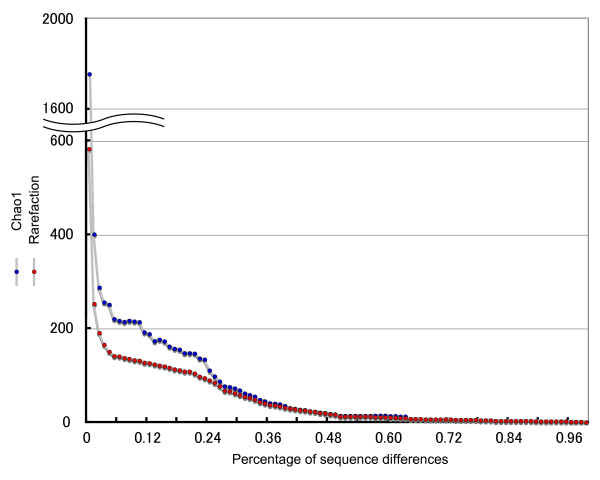
**Relationship between species richness estimated by Chao1, rarefaction, and the percentage sequence difference used for these estimations**.

A zooplankton sample was collected off Pohnpei Island, Micronesia (6°16'N, 162°09'E). A cDNA mitochondrial *COI *(*cytochrome *c *oxidase subunit I*) gene library was constructed from the sample, and 1,336 inserts containing the mitochondrial *COI *gene were randomly sequenced [DDBJ: AB332438-AB333773]. A cDNA rather than a gDNA library was constructed to remove pseudogene sequences from the analysis [4]. The mismatch distribution of these 1,336 sequences revealed a high frequency of very small (<0.03) genetic distance sequence pairs (Figure 1). These sequence pairs with very small genetic distances were assumed to have originated from the same species (discussed below). A second peak was observed around a distance of about 0.14 (from 0.13 to 0.16), and most of these counts were comparisons between two phylogroups in the Copepoda clade (Figure 2, Clades 1 and 2). The frequencies between these peaks were very low. The minimum frequency (106 counts) was observed in the range between 0.12 and 0.13. Based on this observation, we set the criterion that if the genetic distance of two sequences was greater than 0.12, the sequences were derived from different species. If the genetic distance of two sequences was less than 0.12, then we considered the sequences to be derived from the same species. The genetic distances of the mitochondrial *COI *gene sequence have been reported from various animal taxa (mainly Vertebrata and Arthropoda), and the general ranges for the intra- and interspecies distances are 0.0001-0.05 and 0.04-0.21, respectively (Kimura two-parameter model) [5]. Although it is not a conclusive value for animal species definition, we have tentatively taken a genetic distance of 0.12 as the boundary between intra- and interspecies distances in this study, and this value was in the range of interspecific genetic distance reported previously [5]. The rarefaction curve was estimated using the criterion of a genetic distance of 0.12 (Figure 3) using DOTUR [6]. Although the number of observed OTUs is still growing in 1,300 sequenced colonies, the rate of increase of the curve decreased gradually after around 100 sequenced colonies. Figure 4 shows the relationships between species richness estimated by Chao1, rarefaction, and percentage of sequence differences used for the estimation. The figure shows gradual changes of Chao1 and rarefaction around sequence differences of 0.12. As the distance of 0.12 is not a conclusive value for species definition, caution is required in the further use of this value.

We conducted BLASTN searches against the GenBank non-redundant database using as queries all sequences derived from the analysis. Among the sequences, those that fulfilled the criteria (BLAST score and similarity greater than 100 and 83%, respectively) were assigned to 11 taxonomic groups (Figure 2). Several of the assigned sequences showed very high degrees of nucleotide similarity to known species, including Copepoda (*Candacia longimana*, *Cosmocalanus darwinii*, *Neocalanus robustior*, *Rhincalanus rostrifrons*), Euphausiacea (*Stylocheiron carinatum*), Mollusca (*Clio pyramidata*, *Sthenoteuthis oualaniensis*, *Strombus mutabilis*, *Strombus wilsoni*), and Vertebrata (*Coryphaena hippurus*; Table 1). The very high degrees of similarity indicated that these species were actually collected in our sampling. Among them, one vertebrate species, *Coryphaena hippurus*, and two benthic gastropod species, *Strombus mutabilis *and *Strombus wilsoni*, were sampled as non-holozooplanktonic animals in the dispersal life history phase as pelagic larvae. These observations indicated that application of this analysis enables the estimation of larval dispersal, which is difficult to achieve based on morphological observations.

**Table 1:** BLASTN search results for sequences that showed more than 98% similarity to subject sequences

**Colony Number**	**Accession Number**	**Subject Species**	**Score**	**E-value**	**Identity**	**Similarity (%)**
**Copepoda**						
003_P19	AY145435	*Candacia longimana*	959	0	496/500	99
003_B21	AF462311	*Cosmocalanus darwinii*	902	0	479/487	98
003_L24	AY144465	*Neocalanus robustior*	940	0	486/490	99
010_B17	AY371094	*Rhincalanus rostrifrons*	597	2e-167	307/309	99
**Krill (Euphausiacea)**						
003_E12	AF371987	*Stylocheiron carinatum*	944	0	491/496	98
**Mollusca** 005_F13	DQ238000	*Clio pyramidata*	898	0	468/473	98
004_J12	DQ885841	*Sthenoteuthis oualaniensis*	967	0	497/500	99
012_A13	DQ525218	*Strombus mutabilis*	938	0	485/489	99
012_A19	DQ525214	*Strombus wilsoni*	906	0	481/489	98
**Vertebrata**						
004_G22	DQ027995	*Coryphaena hippurus*	950	0	485/487	99

Figure 2 shows an unrooted neighbour-joining tree of the 1,336 zooplankton *COI *sequences. Overall, each taxonomic group formed a single cluster including Gastropoda, Chaetognatha, Euphausiacea, Decapoda, Vertebrata, Copepoda, and Cephalopoda. There were also two cases in which the taxonomic assignment did not work well. The first was the occurrence of Hexapoda in various clusters, which rarely occurs in the ocean environment, except pleustonic insects of the genus *Halobates*. The second was the difficulty of assignment of taxonomic groups due to low BLAST scores and similarities (coloured grey in Figure 2). The most plausible reason for these ambiguities is the paucity of mitochondrial *COI *sequences for some taxa in the DNA database. In general, the mitochondrial *COI *gene sequences in the DNA database are biased among taxa, and this bias was assumed to be the main reason for the occurrence of Hexapoda in our analysis. The most efficient solution for these problems will be the expansion of zooplankton DNA barcode, and it is hoped that the progress of the Barcode of Life project [7] in collaboration with the Census of Marine Zooplankton will fill these gaps.

To our knowledge, the Discovery SOND cruise [8] is the only other attempt to date to estimate the species richness of a whole zooplankton community collected at a single site. In this series of studies [9-19], a total of 618 species of zooplankton were identified and counted in samples collected around the Canary Islands (Table 2). The extrapolated species richness (Chao1 [20]) of the present study was estimated as 188.90 (95% confidence interval, 156.79-255.60) using DOTUR [6]. Our results cannot be directly compared with the SOND cruise data because of differences in sampling effort between the two studies. In the SOND cruise, two primary types of sampling equipment were used: Isaacs-Kidd midwater trawl and N113H.

**Table 2:** Comparison of species that occurred in the present study and the SOND cruise

**Taxa**	**Present Study (%)**	**SOND Cruise (%)**
Amphipoda		106 (17.2)
Cephalopoda	4 (2.1)	18 (2.9)
Copepoda	81 (42.2)	220 (35.6)
Decapoda		35 (5.6)
Euphausiacea		28 (4.5)
Gastropoda	8 (4.2)	
Ostracoda		35 (5.7)
Siphonophora		64 (10.4)
Vertebrata	11 (5.7)	112 (18.1)

Total	192.47	618

Furthermore, about 76 vertically stratified zooplankton samples that were collected above 1,000 m were combined to estimate the occurrence of species [19]. In contrast, the present study was conducted based on a single sample collected from a depth of 721 m to the surface. These sampling effort differences may have accounted for the differences in species richness between the SOND cruise and the present study. In addition, the lower species richness in the present study may have been due to our experimental design. In the present study, after construction of the cDNA library from mRNA, the mitochondrial *COI *genes were amplified with "universal (LCO1490 [21])" and polyT primers. It is possible that some of the mitochondrial *COI *gene sequences may not have been amplified due to primer mismatch for some species. Although the single-gene zooplankton community analysis approach is an efficient means of collecting sequence information, given technical difficulties due to primer mismatch, further studies and the development of novel methodologies are required to gain a complete understanding of zooplankton diversity.

## Conclusion

Although the estimation of species richness and composition of the community are among the most important aspects of single-gene zooplankton community analysis, these sequence data will be further utilised by construction of a dedicated database. We expect that the accumulation of additional marine animal mitochondrial *COI *gene sequence data in the barcode project will aid in further clarifying sequences from unknown species. Furthermore, this process of sequence assignment to particular species through database analysis indicated the occurrence of these species in the sampling site for the present study. We have now constructed a publicly accessible zooplankton community analysis database that can be searched using BLASTN [22].

With regard to the future of zooplankton community genetic analysis, adoption of next-generation sequencing technology should enable researchers to read libraries sufficiently to estimate species richness without extrapolation [23,24]. We are currently expanding our sampling effort to all oceans to further understand zooplankton biodiversity.

## Methods

### Zooplankton sampling

The sample was collected off Pohnpei Island, Micronesia (6°16'N, 162°09'E). Collection was performed with a plankton net (ORI net [25]) with a 2-m^2 ^mouth opening and 0.69-mm mesh aperture. After removal of large animals (more than about 4 cm at their largest measurement), the sample was split into two fractions: one was preserved in ethanol for barcode analysis and the other was homogenised with TRIZol (Invitrogen) and kept at -80°C. A total wet volume of about 30 mL zooplankton was collected and homogenised with 270 mL TRIZol in this step.

### Total RNA extraction and mRNA purification

In the laboratory, total RNA was extracted from the sample following the TRIZol protocol, followed by mRNA purification using Poly(A)Purist MAG (Ambion). A total of 9.6 mL total RNA (aqueous phase) was further purified for mRNA in this step.

### Mitochondrial COI gene library construction and sequence analysis

The purified mRNA was used as the template for Creator SMART cDNA Library Construction Kit (BD Biosciences). Using this constructed cDNA library, we amplified mitochondrial *COI *genes using *COI *universal (LCO1490) [21] and polyT primers with restriction sites that were further used to construct a mitochondrial *COI *gene library with the same kit. We then randomly analysed colonies obtained on agar plates.

### BLASTN search and taxonomic assignment

The lengths of all obtained sequences were adjusted to 500 base pairs, and a BLASTN [26] search against the NCBI non-redundant dataset with default settings was performed with all sequences as queries. Those sequences that did not show any similarity to the mitochondrial *COI *gene sequences were removed (the search was performed in November 2006). BLASTN search against the NCBI non-redundant dataset was also used to infer species or higher taxonomic groups of mitochondrial COI gene sequences determined in the present study. In the BLASTN result list, the species with the highest score was assigned to each sequence with the following criteria. If the BLASTN score was 100 or more and BLASTN similarity was 98% or more, the name of the resulted species was assigned to the sequence and listed in table 1. If the BLASTN score was 100 or more and BLASTN similarity was 83-98%, the name of higher taxon group to which the resulted species belongs was assigned to the sequence and is shown in the figure 2. If BLASTN scores and similarity values did not reach these values of criteria, 'unknown' was assigned to the sequences and are colored gray in the figure 2.

### Removal of PCR recombination, mismatch distribution analysis, rarefaction curve analysis, phylogenetic analysis

To remove sequences produced by PCR recombination, we manually applied a partial treeing approach [27] to the aligned dataset; although some programs and servers are available for related analysis, none worked appropriately for our analysis. Briefly, after the sequence alignment was adjusted using ClustalX [28], square distance matrixes of both the left 100 and right 100 base pairs of the aligned sequence were constructed in MEGA3.1 [29]. Then total absolute deviations of each sequence in these matrixes were calculated. As a result, we deleted one sequence that showed a very large deviation from the others. We assumed this was not the only chimera sequence that occurred in the analysis, but it was not possible to eliminate all PCR recombination sequences because of ambiguity. After removing the PCR recombination sequences from the analysis, we again adjusted alignment of the remaining 1,336 sequences using ClustalX. An unrooted phylogenetic tree was constructed using the neighbour-joining method with nucleotide *p*-distances (alignment gaps were completely deleted) implemented in PAUP*4.10b [30]. The reliability of each tree node was assessed using the bootstrap method with 1,000 replicates. The mismatch distribution was estimated from the distance matrix. The distance matrix was also calculated using PHYLIP3.66 [31], and the matrix was further used for rarefaction curve and Chao1 calculation using DOTUR [6].

## Authors' contributions

R.J.M. was responsible for most of the project planning and experiments other than the BLASTN searches, which were performed by Y.H., M.N., and S.N., who were also responsible for directing the laboratories. The manuscript was prepared by R.J.M. and revised by all other authors.
